# Speaking to nature: a deep learning representational model of proteins ushers in protein linguistics

**DOI:** 10.1093/synbio/ysz013

**Published:** 2019-05-21

**Authors:** Daniel Bojar

**Affiliations:** 4058 Basel, Switzerland

Understanding, modifying and designing proteins require an intimate knowledge of their 3D structure. Even structure-agnostic protein engineering approaches, such as directed evolution, are limited in scope because of the vast potential sequence space and the epistatic effects that multiple mutations have on protein function. To overcome these difficulties, a holistic understanding of sequence–structure–function relationships has to be established. In their recent preprint, members of the Church Group at the Wyss Institute and collaborators describe a novel approach to predicting protein stability and functionality from raw sequence (1). Their representational model UniRep (unified representation), for the first time, demonstrates an advanced understanding of protein features by means of language modeling.

Using deep learning techniques, which were recently recognized with the prestigious Turing Award, Alley *et al*. built a language model for proteins with amino acids as characters based on natural language processing (NLP) techniques. NLP has not only revolutionized our computational understanding of language—think for instance voice-to-text software—but has been coopted for exciting applications in synthetic biology. The recurrent neural network (RNN; a type of neural network which can process sequential inputs such as text) used by Alley *et al.* was trained by iteratively predicting the next amino acid given the preceding amino acids for the ∼24 million protein sequences contained in the UniRef50 database. The RNN thus gathered implicit knowledge about the context of a given amino acid and higher-level features such as secondary structure. The authors then averaged the protein representation of their RNN at every sequence position to yield a protein language representation they call UniRep. They then extended UniRep by adding representations of the final sequence position of their RNN to generate the more complete representation called ‘UniRep Fusion’, which serves as an overview of the entire protein sequence.

UniRep Fusion was then used as an input for a machine learning model to predict protein stability. Notably, this architecture was more accurate than Rosetta, the *de facto* state-of-the-art for predicting protein stability. Their protein language representation allowed the authors to predict the relative brightness of 64 800 GFP mutants differing in as few as one amino acids. Remarkably, their predicted relative brightness values correlated strongly with experimental observation (*r* = 0.98).

UniRep, as the representation of ∼24 million proteins, captures many phenomena of general importance for protein structure and function. These general features can be complemented by dataset-specific attributes when training on a subset of protein mutants or *de novo* designed proteins. This approach could for instance be adopted for screening novel proteins generated by deep learning models. Analogous to *de novo* designed proteins by Rosetta, generating proteins through protein language models might be most advantageous for proteins with radically new functionalities, which are unlikely to be generated by incremental directed evolution.

To arrive in this virtual world of protein engineering though, more advances have to be made. It required the authors of UniRep 1 week of GPU usage to train their large model for one epoch (seeing every protein sequence in UniRef50 once). Switching from the redundancy-ridden UniRef50 database (∼24 million sequences) to preUEP (2), a redundancy-reduced protein sequence database (∼8 million sequences), might enable faster training. This reductionist approach might allow for the ‘vocabulary’ of the model to be extended from single amino acids to larger protein fragments, capturing more structural properties. In general, there are a plethora of NLP techniques developed for written languages which might be useful in protein linguistics. One particularly promising concept would be attention (3), the selective focus on sequence stretches far away from each other, which dramatically improves language models. Given that protein language may be considered one of the most natural languages by definition, modern NLP techniques could transform protein linguistics into a potent tool for the study as well as engineering of proteins for the purposes of synthetic biology.
